# Assessment of Instability in Thoracolumbar Burst Fractures Using a New Bone Scan Scoring System

**DOI:** 10.3390/medicina58080979

**Published:** 2022-07-22

**Authors:** Hyung Jin Choi, Seol Hoon Park, Jun Ik Choi, Jae Young Kim, Minjung Seo

**Affiliations:** 1 Department of Nuclear Medicine, Ulsan University Hospital, Ulsan 44033, Korea; 0735987@uuh.ulsan.kr; 2 Department of Nuclear Medicine, Ulsan University Hospital, University of Ulsan College of Medicine, Ulsan 44033, Korea; 79Eureka@gmail.com; 3 Department of Orthopedics, Dongkang Medical Center, Ulsan 44033, Korea; platonbomb@hanmail.net; 4 Department of Radiology, Ulsan University Hospital, University of Ulsan College of Medicine, Ulsan 44033, Korea; naimami@uuh.ulsan.kr

**Keywords:** burst fracture, bone scan, scintigraphy, unstable, TLICS

## Abstract

*Background and Objectives*: Unstable thoracolumbar burst fractures require surgical management as they can result in neurological deficits if left untreated. This study aimed to evaluate whether a new bone scan scoring system could accurately assess instability in thoracolumbar burst fractures. *Materials and Methods*: Fifty-two patients with thoracolumbar burst fractures who underwent bone scans and magnetic resonance imaging prior to surgery between January 2015 and August 2017 at Ulsan University Hospital were selected for inclusion. Instability was determined by clinical assessment and imaging, and the Thoracolumbar Injury Classification and Severity score was determined. Bone scans were visually evaluated using a new bone scan scoring system. Bone scan findings of vertebral body (B_B_) and posterior column (B_P_) were scored separately and were summed to produce B_TS_ {B_TS_ (total score) = B_B_ (body score, 5 points) + B_P_ (posterior score, 2 points)}. The diagnostic performance of the scoring system for identifying unstable then thoracolumbar burst fractures were assessed. *Results*: Of the 52 thoracolumbar burst fractures, 34 (65.4%) were unstable and 31 (59.6%) had a Thoracolumbar Injury Classification and Severity score ≥ 5. The diagnostic performance of using B_TS_ ≥ 4 to identify unstable thoracolumbar burst fractures and those with a Thoracolumbar Injury Classification and Severity score ≥ 5 was as follows: sensitivity, 61.8% and 58.1%; specificity, 94.4% and 81.0%; positive predictive value, 95.5% and 81.8%; and negative predictive value, 56.7% and 56.7%, respectively. *Conclusions*: The proposed bone scan scoring system has a high specificity and positive predictive value for identifying thoracolumbar burst fractures that are unstable or have a Thoracolumbar Injury Classification and Severity score ≥ 5. This scoring system may help to inform decisions regarding surgical management.

## 1. Introduction

Spinal injuries most frequently occur in the thoracic and lumbar regions, especially at the thoracolumbar junction. There is an increasing incidence of thoracolumbar injury worldwide, mainly due to increased fractures of the elderly population in developed countries and increased motor vehicle accidents in developing countries [1]. Of thoracolumbar injuries, 10–20% are classified as burst fractures [2]. Burst fractures can be defined using the popular Denis three-column classification system as those that involve anterior column compression, middle column fracture, and retropulsion of bony fragments into the spinal canal [3]. These fractures usually result from high-energy trauma causing vertical compression of the spine.

The management of thoracolumbar burst fractures (TLBF) remains challenging, and there is substantial controversy concerning the indications for surgery and the choice of surgical approach. In each case, treatment decisions should be based on a full evaluation of the available clinical and radiographic information. The Denis three-column classification system uses the terms “stable” and “unstable” [3]; however, these terms can be ambiguous and the correlation between stability and the need for surgery is not very strong. In 2005, the Spine Trauma Study Group introduced the Thoracolumbar Injury Classification and Severity Score (TLICS) which consists of three parameters: morphology, posterior ligamentous complex, and neurologic involvement. It is designed to assist the clinical management of thoracolumbar injuries as the total TLICS score predicts the need for surgery [4]. The validation of this scale and the estimation of its reliability are still in process, and there is a need for data regarding the wider application of the system across multiple physicians and centers [5]. Overall, medical decision-making in cases of spinal trauma remains controversial. Although there is wide acceptance of the conservative treatment of stable thoracolumbar fractures according to the TLICS system, specific factors, such as age, stage, and etiology, should not be overlooked; for example, post-traumatic fractures of the junctional region in elderly female patients are reported to have a high risk of conservative treatment failure [6].

Acute thoracolumbar fractures are commonly diagnosed by X-ray plain films and often by computed tomography (CT) or magnetic resonance imaging (MRI). Although CT is reported to be more cost-effective than X-ray plain films, it is limited by radiation exposure and the inadequate assessment of spinal cord ligamentous injuries. MRI can provide a superior assessment of soft tissue but its high cost and low accessibility remain a drawback [7]. ^99m^Tc-methylene diphosphonate (MDP) bone scanning is a relatively simple and highly sensitive imaging technique used for the evaluation of benign and malignant bone pathology [8]. It is widely used in the evaluation of fractures, and many studies have validated its utility in assessing compression fractures. However, to the best of our knowledge, no study has yet investigated the value of bone scans (let alone a scoring system) in assessing burst fractures, despite the worldwide usage of these scans over the last 50 years. Therefore, in the present study, we aimed to evaluate bone scan findings in TLBFs and to determine whether these findings can guide clinical decision-making. 

## 2. Methods

### 2.1. Patients

Records of patients who underwent bone scans between January 2015 and September 2017 at Ulsan University Hospital were retrospectively reviewed, and patients who were diagnosed with TLBFs were considered for inclusion. Patients who underwent spinal MRI in addition to a bone scan for the work-up of a traumatic injury were included. Those for whom the bone scan was performed more than 15 days after trauma or who underwent a laminectomy procedure before the bone scan were excluded. The institutional review board of Ulsan University Hospital approved the retrospective use of clinical data in this study (IRB No. 2017-10-003-003). All procedures were conducted in accordance with the ethical standards of the Responsible Committee on Human Experimentation and adhered to the tenets of the Declaration of Helsinki (as revised in 2000).

### 2.2. Indexes Used to Assess the Indications for Surgery

Many different classification systems have been proposed for TLBFs to aid clinical decision-making [3,9,10]. Two indexes were chosen to assess the indications for surgery in the present study: instability and TLICS.

In the current study, an unstable fracture was defined as a fracture with one or more of the following criteria: associated neurological deficits, posterior element injury, anterior vertebral body height loss of greater than 50%, more than 35 degrees of kyphosis, angulation at the thoracolumbar junction greater than 20 degrees, and spinal canal compromise greater than 30% [2]. 

According to the TLICS system, a severity score of 3 or fewer indicates that conservative management is most appropriate, while a score of 5 or more suggests that surgery is indicated [4]. Injuries with a total score of 4 may be treated either conservatively or surgically. A cut-off value of TLICS 5 was evaluated in this study.

### 2.3. Data Analysis

Data concerning age, history of trauma, bone scan and MRI findings, surgical history, clinical findings at the time of admission, treatment method, and clinical follow-up were collected from the medical record registry. 

### 2.4. Bone Scan Assessment

Bone scans were obtained 2 to 3 h after intravenous injection of 740 to 1110 MBq of either ^99m^Tc-MDP or ^99m^Tc-hydroxymethylene diphosphonate (HDP). Image acquisition was achieved using dual-headed gamma cameras.

**Figure 1:**
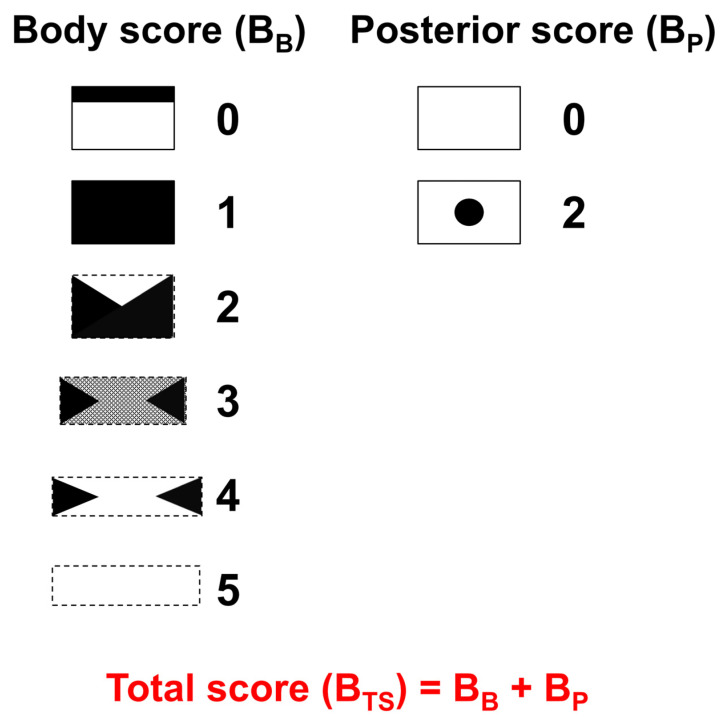
Schematic representation of the new bone scan scoring system which consists of the body score (B_B_) and posterior score (B_P_). B_B_ was classified as follows: 0, linear uptake in the upper endplate; 1, diffuse uptake in the vertebral body; 2, wedge-shaped uptake in the vertebral body; 3, decreased uptake in the central portion of the body but increased uptake bilaterally at the sides; 4, a photon defect in the central portion of the body and increased uptake bilaterally at the sides; and 5, no uptake at all in the body. B_P_ was scored using posterior view images as follows: 0, no uptake; and 2, definite uptake in the spinous process area. The total sum of these scores (B_TS_) was calculated (B_TS_ = B_B_ + B_P_).

Bone scans of TLBFs were evaluated by two experienced nuclear medicine physicians according to the newly proposed bone scan scoring system which is illustrated in Figure 1. When multiple TLBFs were present, the spinal segment with the most severe injury was chosen for evaluation. Bone scan findings regarding the vertebral body (B_B_) and the posterior column (B_P_) were scored separately. B_B_ was scored as follows: 0, linear uptake in the upper endplate; 1, diffuse uptake in the vertebral body; 2, wedge-shaped uptake in the vertebral body; 3, decreased uptake in the central portion of the body but increased uptake bilaterally at the sides; 4, a photon defect in the central portion of the body and increased uptake bilaterally at the sides; and 5, no uptake at all in the body. B_P_ was scored using posterior view images as follows: 0, no uptake; 1, a suspicion of uptake in the spinous process area; and 2, definite uptake in the spinous process area. The total sum of these scores (B_TS_) was then calculated (B_TS_ = B_B_ + B_P_). 

### 2.5. MRI Assessment

MRI scan images that were obtained within 15 days of the bone scan were evaluated by an experienced radiologist. When multiple TLBFs were present, the spinal segment that was assessed on the bone scan was evaluated. The morphology and height of the vertebral body and the integrity of the posterior ligamentous complex were determined.

### 2.6. Clinical Assessment

The clinical assessment of TLBF patients was performed by an experienced orthopedic physician with specialist spinal expertise. Instability and TLICS were determined after reviewing the medical records and images (MRI, X-ray, and if available, CT). 

### 2.7. Statistical Analysis

Receiver operating characteristic (ROC) curve analysis was used to determine the optimal cut-off values for the bone scan scoring system for identifying unstable TLBFs. Fisher’s exact test was performed using the statistical software package SPSS 21.0 (IBM, Armonk, NY, USA). *p* < 0.05 was considered statistically significant. 

## 3. Results

### 3.1. Patient and Lesion Characteristics

**Figure 2:**
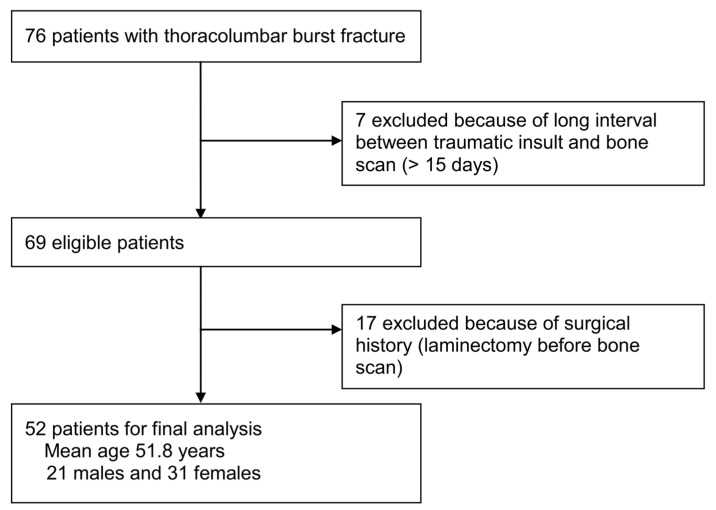
Flow diagram of the selection of patients with thoracolumbar burst fractures. Of the 76 patients who met the inclusion criteria, 7 whose bone scans were performed more than 15 days after trauma, and 17 who underwent laminectomy before scanning were excluded. A total of 52 patients were finally included in the study.

Among the 76 patients who were diagnosed with TLBFs and underwent spinal MRI and a bone scan, 7 patients whose bone scans were performed more than 15 days after trauma, and 17 who underwent laminectomy before scanning were excluded. A total of 52 patients (mean age, 51.8 years; 21 males and 31 females) were included in the study (Figure 2). 

When one lesion (the most severe) was chosen per patient, unstable fractures were found in 65% (34/52) of patients and a TLICS ≥ 5 was observed in 60% (31/52) of patients. Patient and lesion characteristics are presented in Table 1. According to the proposed bone scan scoring system, B_B_ was ≥3 in 60% (31/52) of patients and B_P_ was 2 in 27% (14/52) of patients. The bone scan scoring system results are summarized in Table 2. There was a good interobserver agreement between the two nuclear medicine physicians (κ = 0.75, *p* < 0.001).

### 3.2. Associations between Bone Scan Scoring System Score, Instability, and TLICS

**Figure 3:**
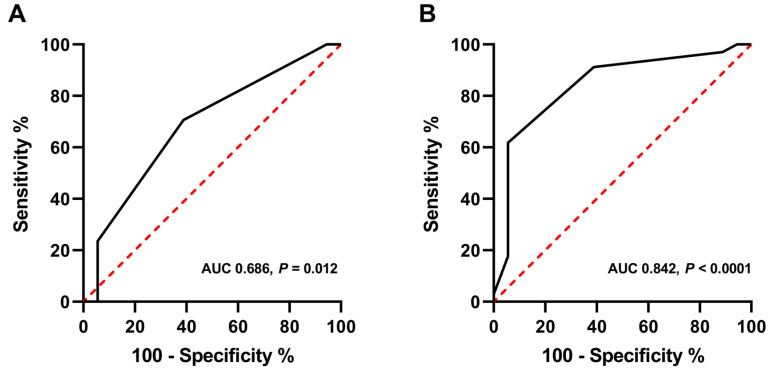
Receiver operating characteristic (ROC) curve analysis of the bone scan scoring system to determine the optimal cut-off values for identifying unstable thoracolumbar burst fractures. B_B_ ≥ 3 and B_TS_ ≥ 4 were found to be the optimal cut-off values. (**A**) The area under the ROC curve (AUC) for B_B_ was 0.686 (95% confidence interval, 0.543–0.808). (**B**) The AUC for B_TS_ was 0.842 (95% confidence interval, 0.714–0.928).

ROC analysis revealed that B_B_ ≥ 3 and B_TS_ ≥ 4 were the optimal cut-off values for identifying unstable TLBFs (Figure 3). The area under the ROC curve (AUC) for B_B_ was 0.686 (95% confidence interval, 0.543–0.808). The AUC for B_TS_ was 0.842 (95% confidence interval, 0.714–0.928). 

With Fisher’s exact test, B_TS_ ≥ 4 was significantly associated with instability and TLICS (instability, *p* < 0.001; TLICS ≥ 5, *p* = 0.009). B_B_ ≥ 3 did not show a significant association with either instability or TLICS (instability, *p* = 0.078; TLICS ≥ 5, *p* = 0.051).

### 3.3. Diagnostic Performance of the Bone Scan Scoring System

**Figure 4:**
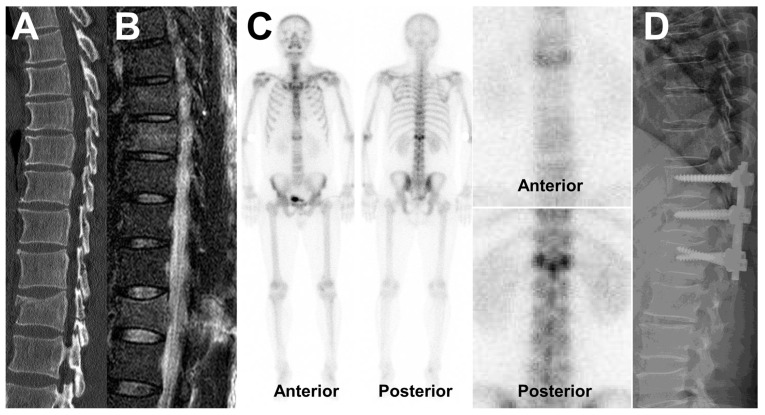
Representative images of a burst fracture of T12 in a 44-year-old patient. (**A**) Computed tomography and (**B**) magnetic resonance images show a T12 burst fracture with damage to the posterior elements of the vertebra. The TLICS was calculated as 7. (**C**) Bone scan images; analysis revealed that B_B_ was 3, B_P_ was 2, and their sum (B_TS_) was 5. The fracture was assessed as unstable and (**D**) the patient underwent posterolateral fusion at the level of T11-L1.

The sensitivity, specificity, positive predictive value, and negative predictive value of B_TS_ ≥ 4 for identifying instability were 61.8%, 94.4%, 95.5%, and 56.7%, respectively. Those of B_TS_ ≥ 4 for predicting TLICS ≥ 5 were 58.1%, 81.0%, 81.8%, and 56.7%, respectively. Images of a representative case are shown in Figure 4.

## 4. Discussion

This study assesses the performance of a newly proposed bone scan scoring system for identifying TLBFs that are unstable or have a TLICS ≥ 5. Unlike previous studies, which have typically been limited to assessing the accuracy of bone scans in vertebral fractures [11,12], the present study proposes a scoring system to aid clinical decision-making when managing patients with TLBFs. To the best of our knowledge, this is the first study to classify the appearance of TLBFs on bone scans according to their severity and to explore the correlation between bone scan findings and TLICS. With regard the proposed bone scan scoring system discussed here, B_TS_ ≥ 4 was found to be the optimal cut-off value.

The overall score on the bone scan scoring system is derived from indexes relating to the vertebral body (B_B_) and the posterior column (B_P_). B_B_, which is scored according to vertebral body uptake, reflects the degree of vertebral body destruction and angulation. B_P_, which is scored according to spinous process uptake, reflects the injury to the posterior column. Since the injury to the posterior column is an important factor to consider when determining the instability of TLBFs, B_P_ was given a weight of 2 in the proposed bone scan scoring system. While a high B_B_ score was not found to be significantly associated with instability or high TLICS, B_TS_, the sum of B_B_ and B_P_, did show a significant association. We, therefore, recommend the use of B_TS_ in the proposed bone scan scoring system. 

Patients with burst fractures require hospitalization and unstable lesions are managed with surgery. These fractures may result in varying degrees of spinal cord injury with possible paralysis, so early detection and determination of instability are important in a clinical setting [2]. These fractures may result in varying degrees of spinal cord injury with possible paralysis; early detection and determination of instability are, therefore, critical to facilitating appropriate interventions to reduce the risk of these sequelae [2]. Assessment of instability is based on clinical and radiological parameters, and efforts to refine various imaging parameters for instability assessment are ongoing. Our study found that a high B_TS_ in TLBFs was significantly associated with instability. When assessing bone scans in patients with burst fractures, it is important to assess the posterior uptake as well as the body appearance to detect instability. 

The TLICS aids in the clinical management of patients with TLBFs; a total score ≥ 5 is often considered to be an indication for surgery [4]. In the present study, B_TS_ ≥ 4 showed a strong association with TLICS ≥ 5, with high specificity. These findings suggest that image analysis using the bone scan scoring system may help to identify patients who do not need surgery. Unfortunately, our study did not include any TLBFs with a TLICS of 4, which are injuries of particular interest to spinal specialists. Currently, there is a lack of consensus on whether surgery is indicated for fractures with a TLICS of 4. One randomized trial conducted by Wood et al. found no significant differences in outcomes between those treated surgically and non-surgically [13], whereas two other randomized trials found better outcomes in surgically treated patients [14,15]. A more recent retrospective study found no significant differences in clinical outcomes between those treated surgically and non-surgically [16]. Further large studies with long-term follow-up data are needed to explore the associations between bone scan findings and clinical outcomes in patients with TLBFs with a TLICS of 4.

Patients who underwent laminectomy prior to bone scan (*n* = 17) were excluded from this study, because postoperative changes may have led to inaccurate scoring on the bone scan scoring system. This led to the exclusion of patients with more severe injuries, leading to the overrepresentation of patients with ‘less severe’ TLBFs. The proposed bone scan scoring system showed good diagnostic performance for these less severe TLBFs, and it could be expected that the actual diagnostic performance across all TLBFs may be even better.

One of the limitations of the present study is that it was retrospective, and data retrieval from the medical records may have been imperfect. Another limitation is that there is a lack of follow-up data since these patients were often later transferred to smaller local centers. Without follow-up data, it is not possible to assess the prognosis of patients with low scores on the bone scan scoring system. The number of assessed TLBFs in this study is relatively small because patients with clearly unstable TLBFs usually undergo an emergent operation. Future prospective studies involving a larger number of patients may further verify the correlation between our new bone scan scoring and the TLICS system and whether a bone scan can help choose the appropriate treatment.

## 5. Conclusions

The proposed bone scan scoring system shows the highest diagnostic performance with a cut-off value of B_TS_ ≥ 4. It has a high specificity and positive predictive value for identifying TLBFs that are unstable or have a TLICS ≥ 5. This scoring system may aid clinical decision-making regarding the surgical management of TLBFs and help to avoid unnecessary surgeries.

## Figures and Tables

**Table 1:** Patient and thoracolumbar burst fracture characteristics (*n* = 52).

Characteristics	*n* (%)
**Sex**	
Male	21 (40%)
Female	31 (60%)
**Age** (years)	Mean 51.8 (range; 20–88)
**Lesion location**	
Thoracic spine	18 (35%)
Lumbar spine	34 (65%)
**Instability**	
Stable	18 (35%)
Unstable	34 (65%)
**TLICS**	
≥5	21 (40%)
<5	31 (60%)

TLICS Thoracolumbar Injury Classification and Severity Score.

**Table 2:** Bone scan scoring system results for included patients (*n* = 52).

Bone Scan Score	*n* (%)
**B_B_** (Body score)	
Score 0	1 (2%)
Score 1	2 (4%)
Score 2	18 (35%)
Score 3	22 (42%)
Score 4	8 (15%)
Score 5	1 (2%)
**B_P_** (Posterior score)	
Score 0	38 (73%)
Score 2	14 (27%)
**B_TS_** (Total score)	
Score 0	1 (2%)
Score 1	2 (4%)
Score 2	11 (21%)
Score 3	16 (31%)
Score 4	15 (29%)
Score 5	6 (11%)
Score 6	1 (2%)

## Data Availability

Not applicable.
